# Dentin tubule occlusion by a 38% silver diamine fluoride gel: an in vitro investigation

**DOI:** 10.1038/s41405-022-00095-8

**Published:** 2022-01-13

**Authors:** Andreas Kiesow, Matthias Menzel, Frank Lippert, Jason M. Tanzer, Peter Milgrom

**Affiliations:** 1 Department of Biological and Macromolecular Materials, Characterization of Medical and Cosmetic Care Products Group, Fraunhofer Institute for Microstructure of Materials and Systems IMWS, Walter-Huelse-Strasse 1, Halle, 6120 Germany; 2 Oral Health Research Institute, Department of Cariology, Operative Dentistry, and Dental Public Health, School of Dentistry, Indiana University, 415 Lansing Street, Indianapolis, IN 46202-2876 USA; 3 Department of Oral Health and Diagnostic Sciences, School of Dental Medicine, University of Connecticut Health, Farmington, CT 06030-1605 USA; 4 Department of Oral Health Sciences, School of Dentistry, University of Washington, Seattle, WA 98195-7475 USA

**Keywords:** Dental materials, Dentistry

## Abstract

**Objective:**

Silver diamine fluoride (SDF) is effective in treatment of dentin hypersensitivity and caries lesions. However, the non-viscous solution does not easily allow clinicians to control the application area. A 38% SDF experiment gel was compared in vitro to commercial SDF for its ability to penetrate and occlude dentinal tubules.

**Materials and methods:**

Human root surface dentin specimens were treated with gelled or standard 38% SDF or negative control. Penetration behavior was established by Drop Shape Analysis. Precipitates at the surface and within tubules were analyzed by SEM and EDX after treatment; Results: penetration depths up to 500 µm were observed for both SDF formulations. Both formulations occluded dentinal tubules similarly. Precipitates on the dentin surface and within dentinal tubules were found for both SDF formulations, with a slight tendency for the experimental gel SDF product to be more abundant than the commercially available one. Discussion: behavior of the experimental 38% SDF gel formulation appeared indistinguishable from the commercial 38% SDF product with respect to dentinal tubule penetration and occlusion.

**Conclusions:**

The experimental 38% SDF gel may be a suitable intervention for the prevention of dentin hypersensitivity.

## Introduction

An expert panel convened by the American Dental Association Council on Scientific Affairs and the Center for Evidence-Based Dentistry has recommended the use of 38% silver diamine fluoride (SDF) as a nonrestorative treatment for carious lesions in the primary and permanent dentitions and in enamel and (root) dentin [1]. As a non-invasive treatment for dentin hypersensitivity, 38% SDF was first introduced into the United States (US) market in 2015. In this context, analyses of affected dentin using scanning electron microscopy and energy-dispersive X-ray spectroscopy have shown that after treatment silver particles could be found on the dentin surface and within the dentinal tubules [2]. The silver particles on the surface and within the tubules decrease tubule diameter markedly and thus reduce tubular fluid movement, an established mechanism to relieve dentin hypersensitivity [3]. Further, the silver is known to help strengthen and harden dentin, and arrest dental caries lesions [4, 5]. In vitro studies show morphological evidence of tubule occlusion [6]; however, open questions remain regarding the mode of action of penetration, precipitation, and the occluding behavior of 38% SDF.

In the US and other countries, 38% SDF is available as an aqueous solution with viscosity similar to water. While there is considerable evidence for its effectiveness in this application form, available formulations have the limitation of being difficult for clinicians to control and excess product can temporarily irritate the gingiva or stain unaffected surfaces. In terms of practicability, a more viscous form of 38% SDF would be advantageous; however, it is important that formulation changes not affect efficacy.

Consequently, we undertook an in vitro study to seek further evidence of the mode of action of 38% SDF in preventing dentin hypersensitivity and compare an experimental gelled 38% SDF formulation to a commercially available 38% SDF product with regard to interactions with dentin.

## Materials and methods

### Design

The study had two parts. First, we determined the penetration behavior of the gelled 38% SDF formulation in comparison to the standard product. Then we conducted a three-arm, blinded, in vitro evaluation of the experimental SDF gel with both placebo solution and positive commercially available SDF control. In the latter in vitro study, there were two primary modes of investigation. Initially, we visualized and quantified open tubules and precipitates on the treated dentin surface. Then, we examined tubule occlusion in fractured cross-sectional samples.

### Specimen preparation, test products and their application

Twenty-eight 4 mm × 4 mm human dentin specimens were prepared at Indiana University from human tooth roots to mimic exposed dentin after gingival recession. Ethical approval for use of the extracted teeth was given by the Indiana University Institutional Review Board (NSO 911-07). The specimens were polished using 1200-grit paper until most of the dentin surface was flattened. The specimens were then serially polished using 4000-grit paper (Struers Inc.) followed by a 1-µm diamond polishing suspension (DP Suspension P, Struers). Highly polished surfaces are preferred to perform the analyses and to relate the findings to those of comparable studies on dentin. While patency of dentinal tubules varies, studies on artificially created, almost- completely-patent tubules are needed to study the mode of action of new interventions and to compare the efficacy between different interventions. Ten untreated specimens were then set aside for the penetration study.

To open the dentinal tubules of the remaining 18 tooth specimens for study of tubule occlusion, all were immersed in 17% EDTA solution (pH 7.4; Fisher Scientific) for 5 min. They were then randomized and allocated 1:1:1 to the test groups before being treated with either one drop (about 50 μL), applied with a microbrush, of an experimental, viscous (~30 cP at room temperature) 38% SDF solution (labeled C, based on Advantage Arrest^®^, patent pending), the positive control (labeled A, Advantage Arrest^®^, Elevate Oral Care LLC), or an aqueous placebo solution that contained neither fluoride nor silver (labeled B), following the manufacturer’s instructions for test group A, the standard product. The SDF solutions were allowed to remain on the exposed surfaces for one minute and then rinsed with running deionized water for 5 s. The test products and placebo were freshly prepared and certified by an FDA-regulated laboratory independent of the investigators, and the investigators were blind to the contents. The specimens were then immersed in artificial saliva (2.2 g/L gastric mucin, 0.381 g/L NaCl, 0.213 g/L CaCl_2._H_2_O, 0.738 g/L KH_2_PO_4,_ 1.114 g/L KCl, pH 7.0; Fisher Scientific) for 2 h [7], rinsed again with running deionized water for 5 s and the vials with excess moisture were sealed and shipped to Germany for testing at the Fraunhofer Institute.

### Penetration behavior

Contact angle measurements were performed using a Drop Shape Analyzer system (DSA 100, Top View Analyzer TVA 100, Krüss, equipped with software KRÜSS ADVANCE 1.10.0.34701). The application of drops was performed in a water vapor-saturated environment at room temperature. Specifically, 0.3 μL drops of each formulation were placed on the sound dentin surface with a micropipette. The kinetics of drop shape change were recorded at intervals of 20 s for 400 s. Since a spreading effect was not observable, a reduction of the contact angle equal to volume reduction was measured. This is expressed as the percent shape/volume decrease. The respective data were averaged and presented with standard deviation for the corresponding time points. Five dentin samples were used for testing each formulation.

### Dentin structure, deposit localization, and deposit identification

Scanning electron microscopy (SEM) was used to characterize the morphology of surfaces and deposits with a SEM-FIB-EDX, Quanta3D FEG Dual-Beam (FEI Company). An acceleration voltage of 10 kV was applied. Energy-dispersive X-ray spectroscopy (EDX) was used to determine the nature of deposits used the Oxford Xplore EDX-Detector (Oxford Instruments) both on surfaces and cross-sectionally. The surface analysis was designed to evaluate deposits formed on the dentin surface region near the dentinal tubules and on the inter-tubular regions. Cross-sectionally fractured samples were used to detect silver deposits within the dentin tubules and to estimate their depth of penetration. The specimens for cross-section analysis were pre-sawed on the backside (opposite site of treatment) to ensure crack initiation. All specimens were coated with an ultra-thin platinum coating before SEM measurements, as customary.

For the surface analysis, nine SEM images were taken randomly at the center of the treated dentin surface and combined using imagery software (cellF, Olympus Soft Imaging Solutions), and then transformed into a binary image (binarized) so that white and black surface elements (white: precipitated silver particles and black: open tubules) could be differentially counted. To decrease the influence of artifacts or undefined surface defects, a size limitation following a normal distribution was applied for the counting of particles. Due to the observational nature of this study, descriptive statistics are presented and expressed as the average percent of closed tubules. The depth analysis in the fractured samples measured the silver penetration, confirmed with EDX, from the dentin surface.

## Results

**Fig. 1:**
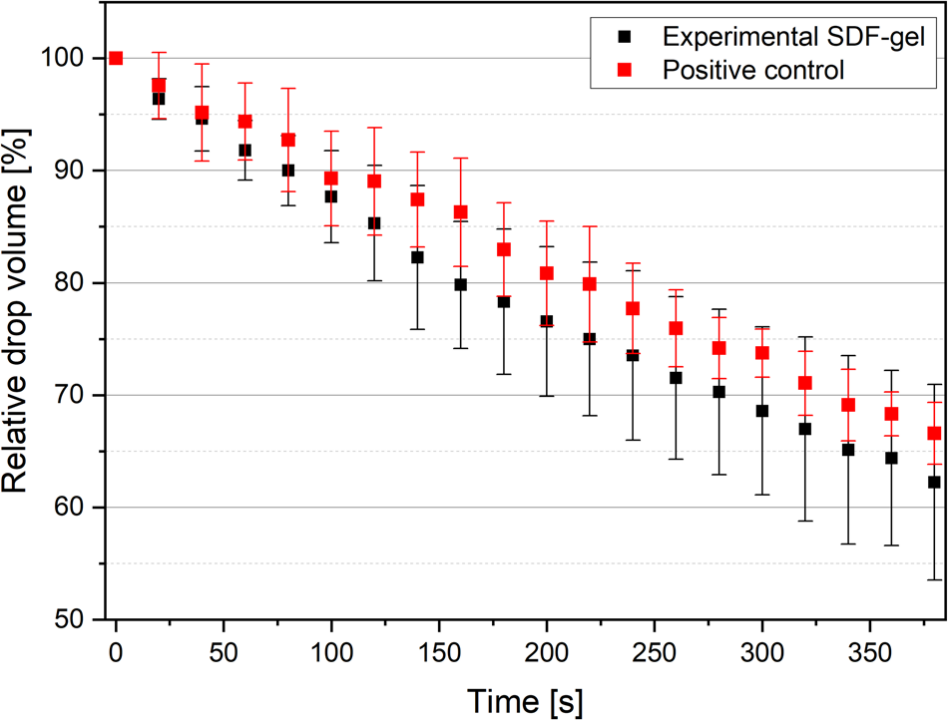
Relative drop volume over time for the experimental SDF gel and positive control. The value of the relative drop volume is related to the respective initial volume of the drop (which is set as 100%).

Figure 1 describes the penetration of the two SDF formulations over time. The drop volume decreased immediately after placing it. The penetration of the two 38% SDF formulations was similar.

### Surface precipitation and tubule occlusion

**Fig. 2:**
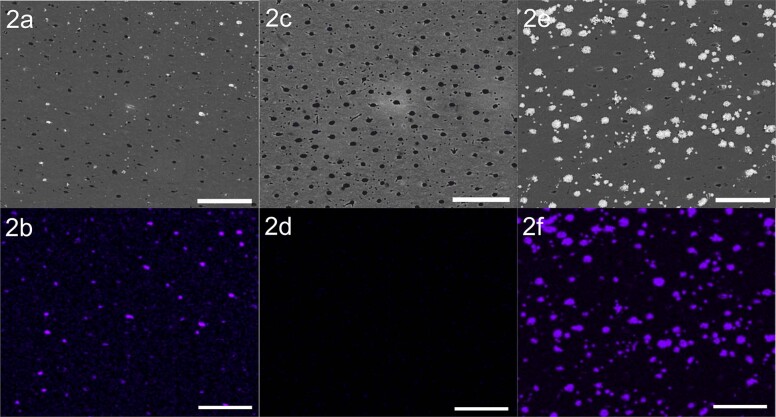
Representative surface imaging by SEM at a magnification of 2500× in backscattered electron mode (material contrast) of treated dentin samples are shown in the upper row. The chemical element mapping is performed using the silver signal. **a**, **b** Show the experimental formulation (sample A2), (**c**) and (**d**) the placebo (sample B2) and (**e**) and (**f**) the positive control (sample C2). The lower row shows element maps of the corresponding SEM-micrographs of upper row. Scale bar = 25 µm.

After application, surface precipitation and tubule occlusion were observed consistently for the positive control (A) and the experimental SDF gel (C) but not for the placebo (B). Representative micrographs are shown in Fig. 2a, c, e. In Figs. 2a, c, the bright structures represent the deposited silver on the dentin surface. The dark structures suggest open tubules. The surface of the placebo-treated dentin is characterized by open dentin tubules. Besides occluded tubules, open tubules are also visible on the dentin surface for groups A and C. EDX-mapping was done on the same samples (Fig. 2b, d, f). Both SEM and EDX images give the impression of slightly higher silver precipitation on the dentin surface for test group C. This was confirmed quantitatively by software-based image analyses: the average percentage of dentin tubules occluded by silver precipitates was 31.4 ± 7.9% for group A and 62.6 ± 24.8% for group C; however, the difference was not statistically significant (*p* = 0.11).

**Fig. 3:**
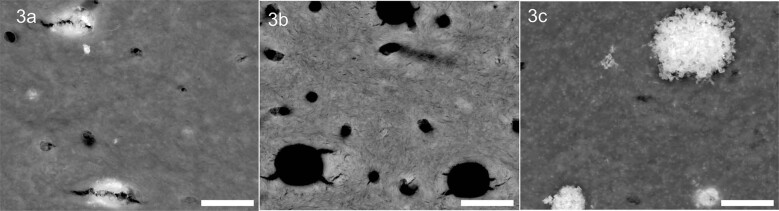
Representative surface images by SEM at a magnification of 25,000× in backscattered electron mode (material contrast) of treated dentin samples (from left: A2, B2, C2). Occluded tubules are visible in 3a and 3c; open tubules are visible in 3c. Scale bar 2.5 µm.

The morphologies of the silver precipitates on the dentin surface differed slightly for the two 38% SDF solutions. Associated with the slightly higher amount of superficially deposited material, the silver precipitates show more elevated structure on the dentin surface after treatment with C (see Fig. 3). In Fig. 3 tubule occlusions as well as openings of tubules with a relatively small diameter are visible on the left (sample A2). Open (seemingly unaffected) tubules are visible in the middle part (sample B2). The SEM micrograph on the right part (sample C2) shows silver precipitates with clearly elevated structure on the dentin surface.

**Fig. 4:**
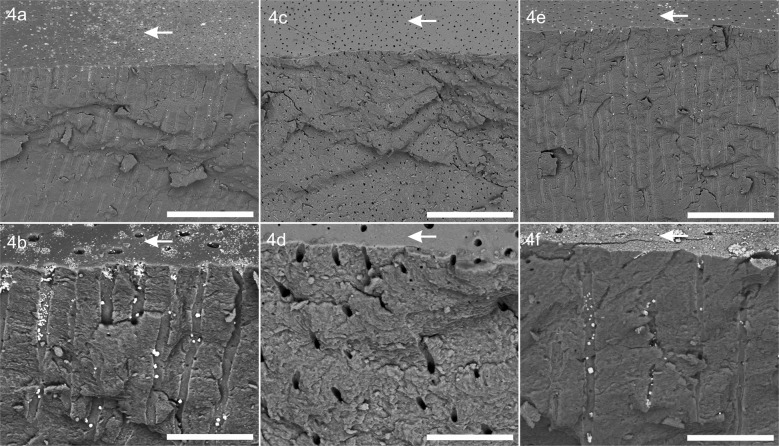
Representative cross section images of fractured dentin samples by SEM in backscattered electron mode: images of the upper row show an overview (scale bar corresponds to 100 µm), whereas images of the lower row depict a detailed view in higher magnification (scale bar corresponds to 20 µm). **a**, **b** Show the experimental formulation (sample A1), (**c**) and (**d**) the placebo (sample B3) and (**e**) and (**f**) the positive control (sample C1). The samples surface is indicated by arrows.

As evident in cross sectional images silver precipitates in form of particles can be observed within as well as at the surface of dental tubules for samples A and C in Fig. 4. Silver particles were not homogeneously distributed within a specimen, as was true for all samples in groups A and C. There were regions within the tubules with higher numbers of particles that appear to form chains of particles, and regions of lower numbers where isolated silver particles were seen. These particulate precipitates were found in almost every tubule, even when there was seemingly no tubule occlusion on the surface. Particle sizes ranged from nearly the diameter of a tubule to far below 1 µm. The number of particles decreased with depth. The depth of penetration of silver particles was measured as up to 500 µm (not depicted by the SEM images). There was no apparent qualitative difference among them in groups A and C. For all B samples, no particulate precipitations were observed within the dentin tubules.

## Discussion

This in vitro study provides evidence for the ability of 38% SDF gel to occlude dentin tubules and thus potentially prevent dentin hypersensitivity and arrest dental caries lesions. Furthermore, the behavior of the experimental 38% SDF gel formulation appeared to be indistinguishable from that of the commercial 38% SDF product.

Despite the differences in viscosity between the test gel product and the commercially available product, there were apparent, impressive differences in the penetration behavior. The data scatter between SEM fields probably can be attributed to the apparent variability of the dentin samples as established probably during tooth formation. The measured volume reduction principally is a result of the penetration of the formulation into the dentin. In the period critical to clinical application the standard SDF formulation and prototype had comparable penetration kinetics.

Tubule occlusion was observed with both the experimental, more viscous form of 38% SDF and the commercially available more liquid 38% SDF product; considerable, although not statistically significant differences were observed between them. However, these differences should be viewed with caution as surface precipitation is possibly of little clinical relevance in the prevention of dentin hypersensitivity. Nonetheless, the greater number of open and wider tubules at the dentin surface could enhance fluid permeability through dentin, and as such increase the possibility for stimulus transmission and subsequent pain response. Because fluid flow is proportional to the fourth power of the tubular radius [8], small changes in tubule diameter likely have a larger effect on fluid flow than might be anticipated. Particulate deposits in the lumens and within the length of the tubules may form a mechanical blockages that could reduce fluid movement inside dentin tubules and thereby alter dentin hypersensitivity [9]. Although the surface analysis suggests that there are a number of seemingly non-occluded tubules, cross-sectional analysis reveals that silver penetrates in almost every tubule and precipitates within them.

Regarding penetration behavior and the resulting particle precipitation, i.e., number, size and distribution of the precipitates within the tubules, both 38% SDF products exhibited comparable penetrations. From a morphological point of view, the particles in general had spherical shape; however, larger particles were more angular, suggesting secondary crystallization processes may occur, similar to what has been reported for insensitive dentin [9].

Penetration depths of about 500 µm were observed for both test products and are in good agreement with previous reports for 38% SDF [2, 10]. However, other studies [6, 11] reported varying degrees of silver penetration into dentinal tubules, perhaps due to differing experimental conditions than used in this study. The morphological characterization of the particles penetrating dentinal tubules was consistent with those near the dentin surface.

The particulate precipitation herein described is consistent with that observed by others [2, 5, 6, 11–14]. The present results contrast, however, with descriptions of “wire-like” structures described by some in their analysis of badly decayed, extracted primary teeth [10]. The decision of these investigators to label the deposited silver within the dentin as “wires” may have been based on the use of synchrotron micro-CT where the spatial resolution is close to that of dentin tubule diameters and likely could not elucidate whether the tubules were completely filled with silver or silver compounds, or whether silver possibly exists in the form of particle chains, as we have observed to some extent (see Fig. 4).

Considering the physical nature of the tubule occlusion on the dentin surface, comparable surface morphologies of the precipitates were observed in other studies [6, 11]. However, Peng et al. [6] have shown that the physical and chemical nature of the superficial precipitates depend strongly on the environment in which the surface reaction between SFD and dentin occurs. Different reaction products or intermediate states have been discussed, such as trisilver phosphate (Ag_3_PO_4_) [13]. Calcium phosphates precipitate with needle shape containing Ca, F, and P or crystals containing Ag and Cl were found by Srisomboon and colleagues [14]. There, the primary mineral precipitation after SDF application is explained by calcium phosphates and silver salts [4, 14]. Further mechanistic considerations of the mode of action are given by Peng et al. [15], for example. It is difficult to determine whether silver, in particular precipitated in the form of particles, is present in metallic form. By EDX analysis, the detection of silver is possible as shown in our study. However, it is not possible to characterize the chemical state of silver by the presently used methods. Other methods, such as X-ray photoelectron spectroscopy (XPS) or transmission electron microscopy (in combination with electron energy loss spectroscopy [EELS]) may be more suitable in this context and be appropriate for future research.

The following limitations must be considered in the interpretation of the present results. This was an in vitro study that cannot comprehensively mimic the complexity of the oral cavity. Nevertheless, dentin samples were chosen to represent the conditions of exposed root surfaces. All measurements were performed almost immediately after SDF application. The protective effect provided by SDF has to be sustained day-to-day against dietary, abrasive, salivary, and other challenges in order to afford persistent benefit. Hence, while the present results are encouraging, they should be confirmed under clinical conditions. Finally, the study compared the experimental gel only to the Advantage Arrest^®^ product. It is not known how it compares to other products that may be on the market in the US or other countries.

## Conclusions

We provided evidence for the ability of an experimental 38% SDF gel to occlude dentin tubules and thus potentially reduce dentin hypersensitivity [3]. Their findings suggest that the actions of the experimental gel product are indistinguishable from the currently available one that was selected for study.

## References

[1] Slayton RL, Urquhart O, Araujo M, Fontana M, Guzmán-Armstrong S, Nascimento MM (2018). Evidence-based clinical practice guideline on nonrestorative treatments for carious lesions: a report from the American Dental Association. J Am Dent Ass.

[2] Li Y, Liu Y, Psoter WJ, Nguyen OM, Bromage TG, Walters MA (2019). Assessment of the silver penetration and distribution in carious lesions of deciduous teeth treated with silver diamine fluoride. Caries Res.

[3] Pashley DH (1990). Mechanisms of dentin sensitivity. Dent Clin North Am.

[4] Mei ML, Lo ECM, Chu CH (2018). Arresting dentine caries with silver diamine fluoride: what’s behind it?. J Dent Res.

[5] Sulyanto RM, Kang M, Srirangapatanam S, Berger M, Candamo F, Wang Y (2021). Biomineralization of dental tissues treated with silver diamine fluoride. J Dent Res.

[6] Peng J-Y, Botelho MG, Matinlinna JP, Pan H-B, Kukk E, Low K-J (2021). Interaction of storage medium and silver diamine fluoride on demineralized dentin. J Int Med Res.

[7] Lippert F, Hara AT (2012). Fluoride dose-response of human and bovine enamel caries lesions under remineralizing conditions. Am J Dent.

[8] Pashley DH, Thompson SM, Stewart FP (1983). Dentin permeability: effects of temperature on hydraulic conductance. J Dent Res.

[9] Yoshiyama M, Masada J, Uchida A, Ishida H (1989). Scanning electron microscopic characterization of sensitive vs. insensitive human radicular dentin. J Dent Res.

[10] Seto J, Horst JA, Parkinson DY, Frachella JC, DeRisi JL (2020). Enhanced tooth structure via silver microwires following treatment with 38 percent silver diamine fluoride. Pediatr Dent.

[11] Willershausen I, Schulte D, Azaripour A, Weyer V, Briseño B, Willershausen B (2015). Penetration potential of a silver diamine fluoride solution on dentin surfaces. An ex vivo study. Clin Lab.

[12] Siqueira F, Morales L, Granja M, de Melo BO, Monteiro-Neto V, Reis A (2020). Effect of silver diamine fluoride on the bonding properties to caries-affected dentin. J Adhesive Dent.

[13] Rosenblatt A, Stamford TC, Niederman R (2009). Silver diamine fluoride: a caries “silver-fluoride bullet”. J Dent Res.

[14] Peng JJ-Y, Botelho MG, Matinlinna JP (2012). Silver compounds used in dentistry for caries management: a review. J Dent.

[15] Srisomboon S, Kettratad M, Pakawanit P, Rojviriya C, Phantumvanit P, Panpisut P (2021). Effects of different application times of silver diamine fluoride on mineral precipitation in demineralized dentin. Dent J (Basel).

